# The role of CD36 in immune function: bridging innate and adaptive responses

**DOI:** 10.3389/fimmu.2026.1728509

**Published:** 2026-01-27

**Authors:** Xiang Zhang, Ling-jie Luo, Yan-wei Wu, Liang Chen

**Affiliations:** 1 School of Medicine, Shanghai University, Shanghai, China; 2 State Key Laboratory of New Targets Discovery and Drug Development for Major Disease, Xi'an, China; 3 Shanghai Tenth People’s Hospital, Shanghai, China; 4 Institute of Artificial Intelligence and Biomanufacturing, School of Medicine, Shanghai University, Shanghai, China

**Keywords:** adaptive immunity, CD36, immune modulation, inflammation, innate immunity, phagocytosis

## Abstract

CD36 is a multifunctional glycoprotein essential in fatty acid metabolism, angiogenesis, and atherogenesis, playing a critical role in immunological processes. This comprehensive review synthesizes current research to elucidate CD36’s integral functions within the immune system, including its involvement in phagocytosis, inflammation, and the crucial interplay between innate and adaptive immune responses. We highlight novel insights into CD36 as a therapeutic target, presenting recent advances in targeting strategies for a spectrum of conditions such as inflammatory diseases, infections, metabolic disorders and cardiovascular diseases. By evaluating emerging research and clinical trials, this review proposes innovative approaches for exploiting CD36’s therapeutic potential, aiming to inspire further research and development in disease treatment.

## 1 Introduction

The immune system is a highly coordinated network of cells, receptors, and signaling pathways that preserves physiological homeostasis and defends the host against pathogens. Central to its effectiveness is the interplay between innate and adaptive immunity, which relies on a wide range of receptors capable of recognizing lipids, metabolites, pathogens, and cellular debris (1). Among these, scavenger receptors have emerged as key mediators, bridging lipid metabolism, immune surveillance, and inflammatory regulation (2).

CD36, a multifunctional glycoprotein initially identified in the 1980s as a receptor for oxidized low-density lipoprotein (oxLDL) in atherosclerosis (3), has since been recognized as far more than a lipid scavenger. It exerts pleiotropic functions in angiogenesis (4), adipocyte lipid storage (5), taste perception (6), and immune regulation (7). Unlike other scavenger receptors such as SR-A (Scavenger Receptor Class A), which mainly clears cellular debris (8), or LOX-1 (Lectin-like Oxidized LDL Receptor 1), which is primarily associated with cardiovascular pathology (9), CD36 demonstrates remarkable ligand promiscuity and signaling versatility. These features enable it to govern diverse immune processes, including the phagocytosis of apoptotic cells and pathogens (10), the activation of antigen-presenting cells (11), and modulation of inflammatory cascades. Through these mechanisms, CD36 functions as a critical molecular bridge between innate and adaptive immune responses.

Importantly, CD36 plays a paradoxical role in immunity: it can either amplify inflammation, contributing to tissue damage and chronic disease, or promote resolution and homeostasis depending on context. This duality has attracted growing interest in CD36 as a therapeutic target in inflammatory disorders, infectious diseases, metabolic syndromes, and cardiovascular conditions. In this review, we synthesize current evidence on the immunological functions of CD36, highlight its role at the intersection of innate and adaptive immunity, and discuss the translational potential of targeting this receptor for therapeutic intervention.

## 2 CD36: structure, expression, and ligand diversity

### 2.1 Protein structure and family background

CD36 belongs to the class B scavenger receptor family, which also includes SR-BI (SCARB1) and SR-BII. It is a heavily glycosylated transmembrane glycoprotein of ~88 kDa, encoded by the CD36 gene on chromosome 7q21.11 (12). Structurally, CD36 is characterized by two short cytoplasmic tails (N- and C-terminus) connected by two transmembrane domains, which flank a large extracellular loop of ~400 amino acids. This extracellular domain contains multiple N-linked glycosylation sites and hydrophobic pockets that confer broad ligand-binding specificity (13). Palmitoylation of the cytoplasmic tails contributes to receptor trafficking, membrane localization, and signal transduction. Functionally, CD36 serves as a pattern recognition receptor (PRR), linking lipid metabolism and immune surveillance, and its structural versatility underlies its ability to interact with diverse ligands ranging from lipoproteins to microbial components (14, 15).

### 2.2 Cellular and tissue distribution

CD36 is widely expressed in metabolically active and immune-relevant tissues. High expression is found in adipocytes, macrophages, dendritic cells, platelets, endothelial cells, cardiomyocytes, and hepatocytes, among others (16). This broad distribution reflects its multifaceted functions: in parenchymal cells such as hepatocytes, adipocytes, and cardiomyocytes, CD36 acts as a fatty acid translocase that supports long-chain fatty acid uptake and energy metabolism; in endothelial cells it regulates angiogenesis and vascular integrity; and in immune cells it couples lipid sensing/uptake to functions such as pathogen recognition, phagocytosis, antigen presentation, and metabolic reprogramming (17). Notably, tissue-specific expression is tightly regulated by transcription factors such as PPARγ, LXR, and nf-Kb, allowing CD36 to adapt its function according to metabolic and inflammatory contexts (18). A detailed summary of cell type–specific expression, ligands, and functions is presented in **Table 1**.

**Table 1:** CD36 expression, ligands, and functions across cell types.

Cell type	Major ligands	Primary functions	Disease relevance
Macrophages	oxLDL, apoptotic cells, PAMPs, AGEs, TSP-1	Foam cell formation, phagocytosis, inflammasome activation, cytokine production	Atherosclerosis, diabetes, chronic inflammation (19)
Dendritic cells (DCs)	Apoptotic cells, microbial ligands	Antigen uptake and presentation, T-cell priming, immune tolerance	Autoimmunity, infection (20)
Neutrophils	oxLDL, microbial components, phospholipids	Phagocytosis, NETosis, ROS production, cytokine secretion	Cancer progression (21)
Adipocytes	Long-chain fatty acids, lipoproteins	Fatty acid uptake, lipid storage, metabolic regulation	Obesity, metabolic syndrome (22)
Endothelial cells	oxLDL, thrombospondin-1	Angiogenesis regulation, vascular inflammation, apoptosis control	Cardiovascular disease, cancer angiogenesis (23)
Platelets	oxLDL, TSP-1	Platelet activation, thrombosis, vascular remodeling	Thrombosis, cardiovascular events (24)
Cardiomyocytes	Long-chain fatty acids	Energy supply via fatty acid oxidation, metabolic stress response	Heart failure, ischemic heart disease (25)
Taste receptor cells	Long-chain fatty acids	Fatty acid sensing, taste perception	Obesity, dietary preference, metabolic disorders (26)
Hepatocytes	Long-chain fatty acids, oxLDL	Hepatic fatty acid uptake and lipid handling; regulation of lipid storage/oxidation	NAFLD/NASH, insulin resistance (27)

### 2.3 Ligand repertoire and recognition mechanisms

A defining feature of CD36 is its promiscuous ligand-binding capacity. It recognizes a wide spectrum of endogenous and exogenous molecules, enabling it to act at the intersection of lipid metabolism, immunity, and inflammation. Endogenous ligands include long-chain fatty acids, oxLDL, high-density lipoproteins (HDL), thrombospondin-1, and advanced glycation end products (AGEs). Exogenous ligands encompass pathogen-associated molecular patterns (PAMPs), such as components of bacterial cell walls and parasitic molecules. Recognition of apoptotic cells is mediated through exposure of phosphatidylserine and oxidized phospholipids on dying cell membranes (28).

Mechanistically, ligand binding triggers conformational changes in CD36 that recruit intracellular signaling adaptors, such as Src family kinases, JNK, and MAPKs, thereby initiating downstream pathways that regulate phagocytosis, cytokine production, and metabolic reprogramming (29). Importantly, CD36 often cooperates with Toll-like receptors (e.g., TLR2/6) to amplify inflammatory signaling, while in other contexts it promotes anti-inflammatory and homeostatic responses (30). This ligand diversity and context dependence explain the dualistic roles of CD36 in disease progression and resolution.

## 3 Historical and functional evolution of CD36

The scientific understanding of CD36 has undergone remarkable evolution over the past five decades, transitioning from its initial identification as a platelet surface protein to its recognition as a multifunctional receptor with broad physiological and pathological relevance.

### 3.1 1970s–1980s: platelet glycoprotein, oxLDL uptake, and atherosclerosis

CD36 was first identified in the late 1970s as a glycoprotein on the surface of platelets, later named glycoprotein IV (31). By the early 1980s, it was established that CD36 played a critical role in the uptake of oxLDL, linking it to the development of foam cells and atherosclerosis (32). This discovery positioned CD36 as a key factor in lipid metabolism and cardiovascular diseases.

### 3.2 1980s–1990s: angiogenesis, adipogenesis, and immune modulation

In the following decades, CD36 research expanded beyond lipid handling to encompass diverse biological processes. Studies in the 1990s identified its function in angiogenesis through interactions with thrombospondin-1, whereby CD36 mediated anti-angiogenic signaling in endothelial cells (33). Concurrently, CD36 was shown to regulate fatty acid uptake and lipid storage in adipose tissue, highlighting its role in adipogenesis and energy homeostasis (34). Additionally, CD36 emerged as a key immune modulator, mediating phagocytosis of apoptotic cells, recognizing microbial ligands, and interacting with Toll-like receptors in innate immunity (35).

### 3.3 1990s–2000s: expanding roles in metabolism and inflammatory disease

During the 1990s and 2000s, CD36’s functions in metabolism and immune modulation further expanded. It was shown to regulate fatty acid uptake in tissues like muscle and liver, emphasizing its role in metabolic disorders (36). CD36’s involvement in immune responses was clarified, particularly in phagocytosis and receptor interactions, enhancing both innate and adaptive immunity. The receptor was increasingly linked to chronic inflammatory diseases like atherosclerosis and osteoarthritis, where its interaction with oxidized lipids contributed to disease progression (37). Its association with metabolic disorders such as obesity and insulin resistance highlighted its potential as a therapeutic target.

### 3.4 2000s–present: sensory perception, metabolic disease, cancer, and immunity

Since the 2000s, CD36’s functional repertoire has expanded further. Notably, it was found to mediate the oral detection of long-chain fatty acids, linking it to taste perception. Its dysregulation has been implicated in metabolic disorders like obesity and non-alcoholic fatty liver disease (38, 39). CD36 also plays a significant role in cancer biology, contributing to tumor angiogenesis, lipid-driven metabolic adaptation, and metastatic potential. Additionally, it remains a versatile immune receptor, bridging innate and adaptive responses, making it a key target for therapeutic interventions across various diseases (40–42).

## 4 CD36 in innate immunity

### 4.1 Phagocytosis and efferocytosis: mechanisms and signaling

**Figure 1:**
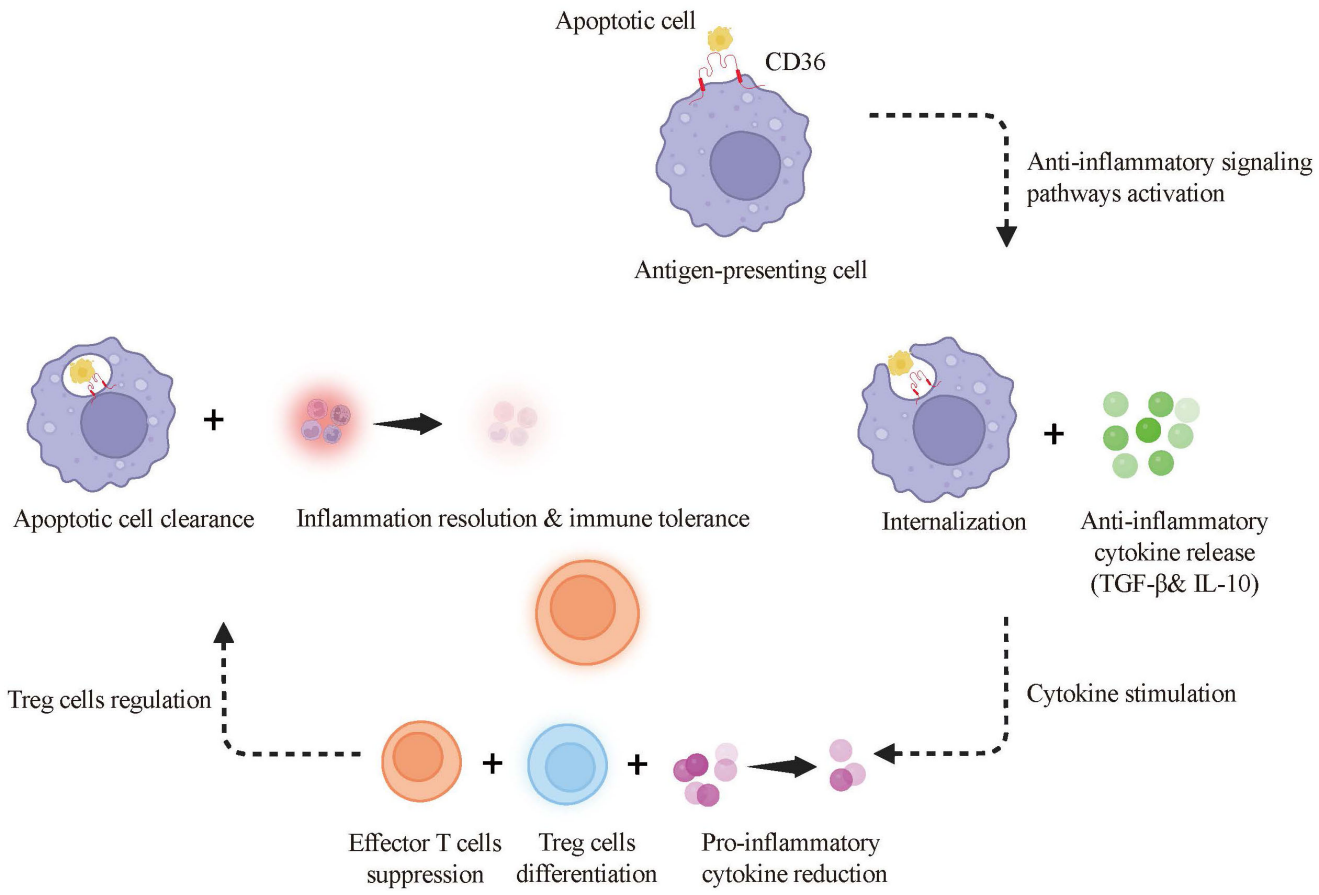
CD36’s role in efferocytosis and immune tolerance. CD36 recognizes and engulfs apoptotic cells by binding to phosphatidylserine on their surfaces, facilitating their internalization. This interaction activates anti-inflammatory signaling pathways, leading to the release of Transforming Growth Factor-Beta (TGF-β) and Interleukin-10 (IL-10). These cytokines suppress effector T cell proliferation, support regulatory T cell differentiation, and reduce pro-inflammatory cytokine production, thereby promoting immune tolerance and resolving inflammation.

Phagocytosis and efferocytosis enable macrophages and DCs to remove pathogens, apoptotic cells, and debris, thereby supporting host defense and tissue homeostasis. CD36 contributes to both processes by recognizing apoptotic cell–associated ligands and microbial-associated patterns, promoting cargo clearance (43). Efficient efferocytosis is particularly important for preventing secondary necrosis, limiting inappropriate immune activation to self-antigens, and reducing autoimmune risk (44–46) (**Figure 1**).

**Figure 2:**
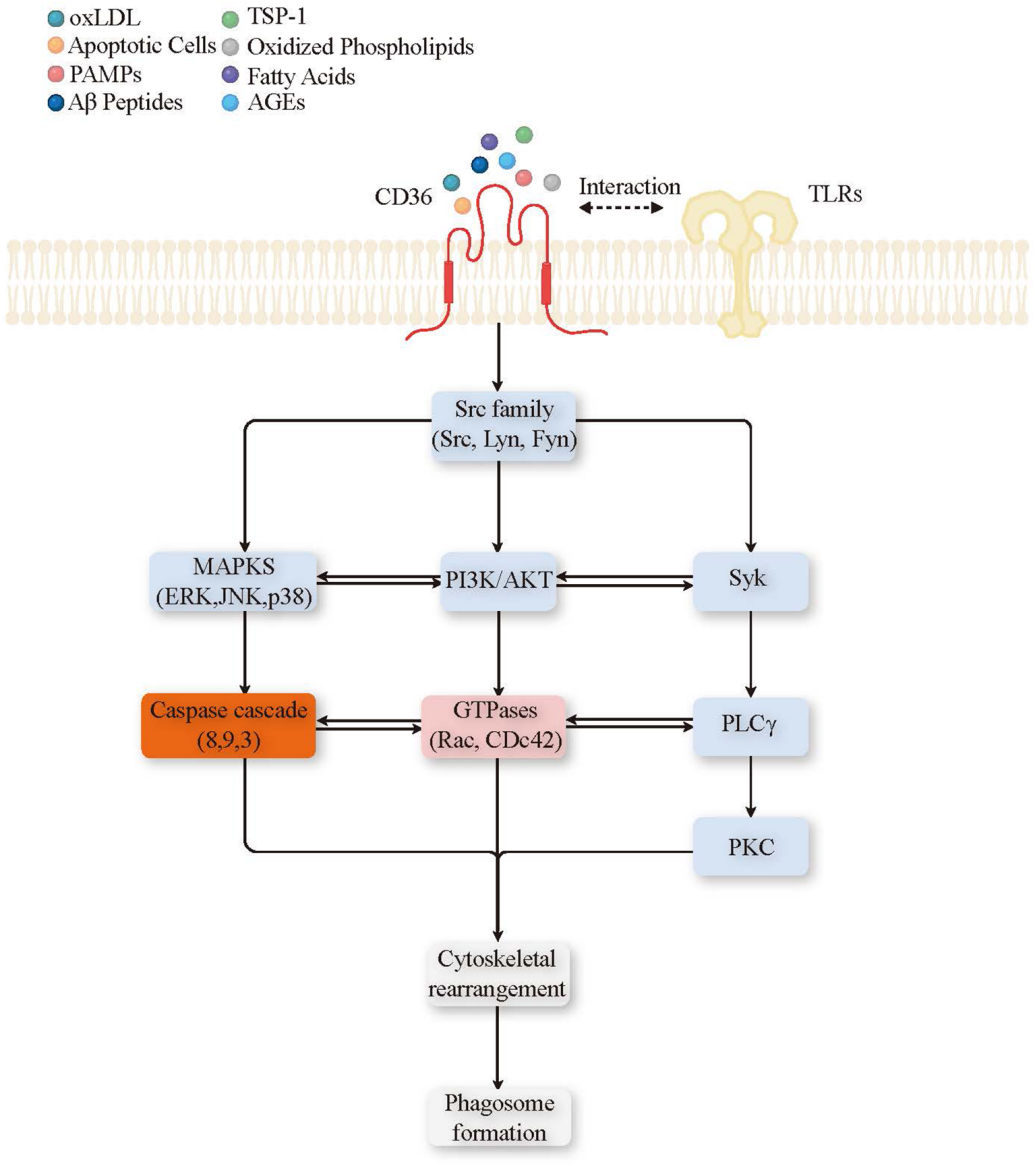
CD36 ligand and signal transduction pathways leading to phagocytosis. Binding of various ligands (oxLDL, TSP-1, PAMPs, Aβ peptides, apoptotic cells, oxidized phospholipids, fatty acids, AGEs) to CD36 on the plasma membrane triggers the recruitment of TLRs, forming a complex that initiates several downstream signaling pathways. Key signaling components include kinases and signaling enzymes such as the Src family (Src, Lyn, Fyn), PI3K/AKT, MAPKs, Syk, PLCγ, and PKC (blue); second messengers and reactive molecules like GTPases (Rac, Cdc42) (pink); inflammatory mediators including the caspase cascade (3, 8, 9) (orange). These pathways converge to facilitate cytoskeletal rearrangement and phagosome formation, thereby promoting phagocytosis. Oxidized Low-Density Lipoprotein (oxLDL), Thrombospondin-1 (TSP-1), Pathogen-Associated Molecular Patterns (PAMPs), Amyloid-Beta (Aβ), Advanced Glycation End-products (AGEs), Non-receptor Tyrosine Kinases (Src family), Proto-Oncogene Tyrosine-Protein Kinase Src (Src), Tyrosine-Protein Kinase Lyn (Lyn), Proto-Oncogene Tyrosine-Protein Kinase Fyn (Fyn), Phosphoinositide 3-Kinase/Protein Kinase B (PI3K/AKT), Mitogen-Activated Protein Kinases (MAPKs), Spleen Tyrosine Kinase (Syk), Phospholipase C Gamma (PLCγ), Protein Kinase C (PKC), and Guanosine Triphosphatases (GTPases) including Ras-related C3 Botulinum Toxin Substrate (Rac) and Cell Division Control Protein 42 (Cdc42).

CD36 rarely acts alone. Upon ligand engagement, it cooperates with other surface receptors—most notably TLR2/6—to enhance particle binding and coordinate intracellular signaling that drives actin remodeling and engulfment (**Figure 2**). In macrophages, CD36–TLR2 cooperation has been linked to improved bacterial uptake and clearance in several infection models (47), supporting a role for CD36 as an amplifier of innate immune responses.

At the molecular level, CD36 ligation (e.g., by oxLDL, anionic phospholipids, or thrombospondin-1) promotes assembly of signaling complexes at the phagocytic synapse. This includes recruitment of Src-family kinases (e.g., Lyn, Fyn) and downstream activation of Syk, which together support phagocytic cup formation, cytoskeletal rearrangement, and inflammatory gene regulation (48–51). CD36 signaling also engages PI3K/Akt to support phagocyte survival and phagosome maturation, facilitating efficient digestion of internalized cargo (25, 52, 53). Small GTPases (e.g., Rac, Cdc42), along with PLC/PKC and MAPK pathways, further coordinate actin dynamics and phagosomal processing (54–56). In select contexts, CD36-dependent signaling has also been associated with caspase activation, which may contribute to clearance programs during responses to infected or transformed targets (57, 58). Collectively, these pathways position CD36 as a multifunctional regulator linking recognition to uptake, processing, and inflammatory tone.

#### 4.1.1 Reconciling discrepant findings in CD36-dependent phagocytosis and efferocytosis

Although many studies support a facilitating role for CD36, its quantitative importance varies across systems. In injury settings enriched for oxidized lipids and apoptotic parenchymal cells (e.g., chronic kidney injury), CD36 deficiency has been associated with impaired corpse clearance and downstream fibrogenic signaling, consistent with a non-redundant contribution under high cargo-load and inflammatory stress (59). In contrast, in tissues where multiple efferocytosis receptors are co-expressed, CD36 loss can be partially compensated by alternative recognition pathways, preserving overall clearance despite changes in phagocyte composition or activation state (60, 61).

Methodological differences likely explain some of the conflicting results. Studies use different apoptotic targets (e.g., neutrophils vs epithelial cells), vary in whether serum-derived opsonins/bridging molecules are present, and measure different endpoints (binding/tethering, internalization, or phagolysosomal degradation). Because CD36 may mainly affect specific steps in this sequence, its “requirement” can appear stronger or weaker depending on the assay used.

A similar context dependence applies to microbial phagocytosis. CD36 can enhance bacterial uptake and inflammatory responsiveness in certain infections (62), consistent with cooperation with TLR2/6 (63). However, when pathogens are strongly opsonized or when redundant pattern-recognition receptors dominate, host defense may rely more heavily on other PRR pathways, minimizing observable CD36 dependency (64). Together, these data support viewing CD36 as a context-sensitive amplifier—most impactful when its ligands are abundant and redundancy is limited, and less apparent when parallel clearance pathways or strong opsonization can substitute.

### 4.2 Innate immune cell activation and function modulation via CD36

#### 4.2.1 Neutrophil activation: NETosis and pathogen clearance

Neutrophils are frontline innate immune cells that rapidly eliminate pathogens through phagocytosis, oxidative burst, and release of granule enzymes. CD36 expression on neutrophils enhances pathogen recognition and uptake, thereby strengthening early antimicrobial defenses and accelerating pathogen clearance (65). Beyond direct killing, neutrophils shape the inflammatory milieu by producing cytokines and chemokines (e.g., IL-8, TNF-α, MIP-1α) that recruit and activate additional immune cells; CD36 engagement can modulate this secretory program, supporting coordinated amplification of the immune response (66, 67).

CD36 has also been linked to neutrophil extracellular trap (NET) formation, a defense mechanism in which chromatin fibers decorated with histones and antimicrobial proteins immobilize and neutralize microbes (68, 69). In several settings, CD36–ligand interactions promote NETosis by driving NADPH oxidase–dependent ROS generation and activating inflammatory signaling pathways such as MAPKs and nf-Kb (70–72), potentially in part through broader effects on cytokine output (73, 74). However, NET release is not uniformly CD36-dependent: robust NETosis can be triggered by stimuli such as PMA or immune complexes via pathways that bypass CD36 (e.g., PKC activation or Fc receptor signaling), reducing the apparent requirement for CD36 (75). Discrepant conclusions across studies likely reflect both stimulus dependence (receptor-proximal vs bypass signaling) and measurement differences, as NETosis is quantified using distinct endpoints (extracellular DNA, citrullinated histone H3, MPO–DNA complexes, or functional microbial trapping) that capture different stages of the process. Using the same stimuli and NET assays in human and mouse experiments will clarify whether CD36 is required for NET formation or mainly affects its magnitude.

#### 4.2.2 Macrophage polarization: metabolic and inflammatory switching

As a fatty acid translocase and scavenger receptor, CD36 links macrophage lipid uptake to immunometabolic remodeling. Uptake of long-chain fatty acids and oxidized lipids via CD36 promotes lipid droplet accumulation and can fuel mitochondrial β-oxidation or ROS generation, thereby shaping inflammatory signaling and foam-cell biology in lipid-rich tissues such as atherosclerotic plaques (76). In addition to lipid uptake, CD36 modulates macrophage polarization. Engagement of CD36 by oxLDL, fatty acids, or AGEs promotes M1-like pro-inflammatory polarization, driving secretion of IL-1β, IL-6, and TNF-α (77). Conversely, CD36-mediated clearance of apoptotic cells favors an M2-like phenotype, characterized by anti-inflammatory cytokine production and tissue repair (78). This context-dependent regulation highlights CD36 as a molecular switch influencing macrophage function in metabolic and inflammatory settings.

#### 4.2.3 Dendritic cell surveillance

CD36 also supports the uptake and clearance of apoptotic cells and microbial ligands by DCs, reinforcing their role in innate immune surveillance. Through this activity, CD36 helps maintain tissue homeostasis and prepares antigens for subsequent presentation to the adaptive immune system (7).

## 5 CD36 in adaptive immunity

While CD36 is known for its critical contributions to innate immunity, it also plays a vital role in shaping adaptive immune responses. By influencing antigen processing, cytokine production, and the activation of antigen-presenting cells (APCs), CD36 bridges the innate immune system to adaptive immunity, influencing T and B cell responses.

### 5.1 Antigen presentation by dendritic cells and macrophages

**Figure 3:**
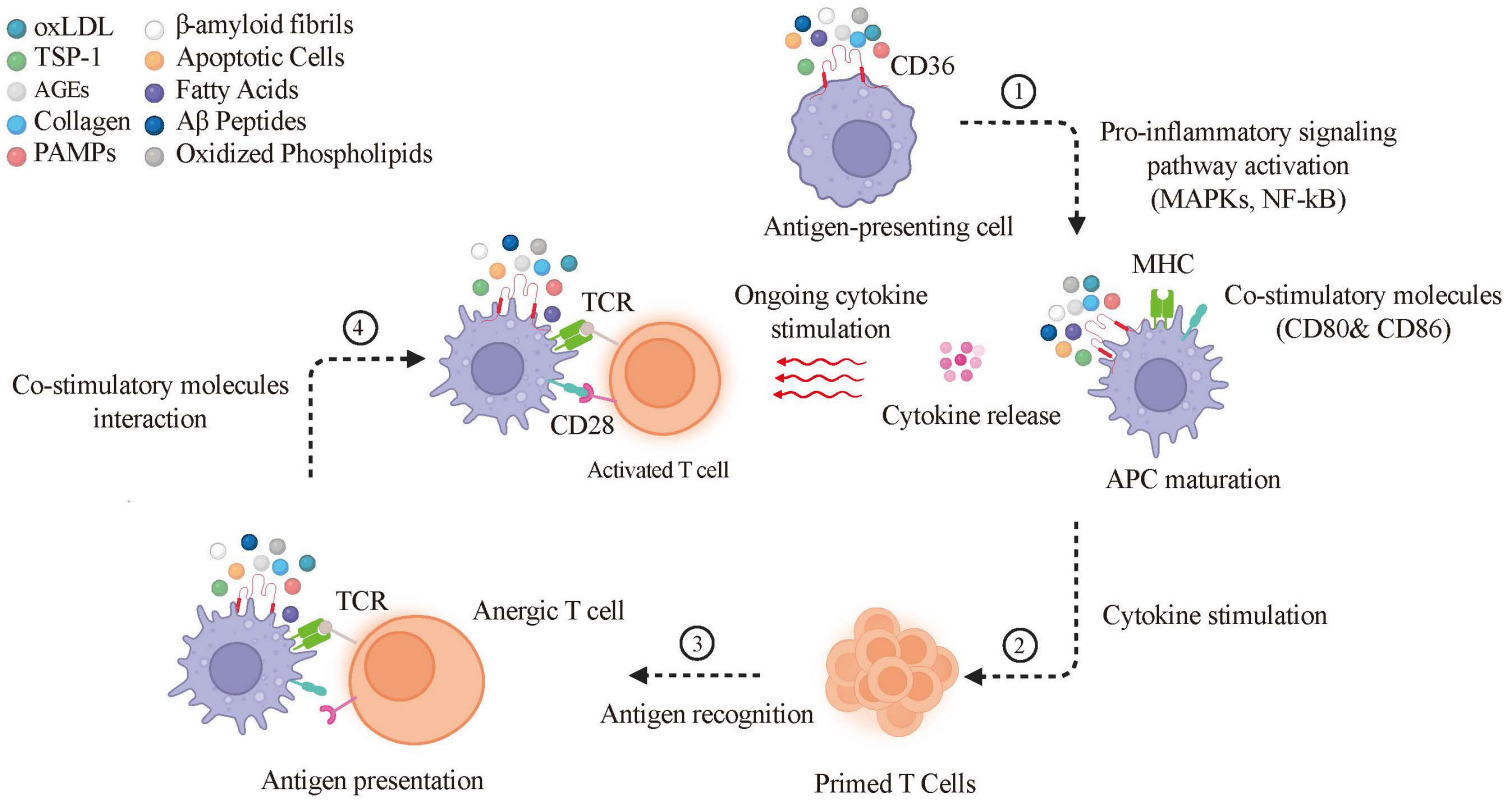
CD36’s role in maturation of dendritic cells and macrophages and subsequent T-cell activation. CD36 interaction with diverse ligands on APCs (such as oxLDL, TSP-1, AGEs, and apoptotic cells) activates MAPKs (ERK, JNK, p38) and the nf-Kb pathway, leading to the upregulation of MHC and co-stimulatory molecules like CD80 and CD86. This interaction also triggers cytokine release, which stimulates primed T cells. The TCR on these primed T cells recognizes antigens presented by the MHC; however, without the additional co-stimulatory signal from molecules like CD28 on the APCs, the T cell remains anergic. Full activation of the T cell is achieved when it receives both MHC-mediated antigen presentation and co-stimulatory signals, along with ongoing cytokine stimulation. Oxidized Low-Density Lipoprotein (oxLDL), Thrombospondin-1 (TSP-1), Advanced Glycation End-products (AGEs), Pathogen-Associated Molecular Patterns (PAMPs), Amyloid-Beta Peptides (Aβ Peptides), Antigen-Presenting Cells (APCs), Mitogen-Activated Protein Kinases (MAPKs) which includes ERK, JNK, p38, Nuclear Factor Kappa-Light-Chain-Enhancer of Activated B Cells (nf-Kb), Major Histocompatibility Complex (MHC), Cluster of Differentiation 80 and 86 (CD80 and CD86), T-Cell Receptor (TCR), and Cluster of Differentiation 28 (CD28).

CD36 on dendritic cells and macrophages promotes uptake of apoptotic cells and microbial ligands, thereby supporting antigen processing and presentation. CD36 enhances both MHC class I cross-presentation and MHC class II presentation, expanding the antigen repertoire available for T-cell priming. Engagement of CD36 (e.g., by oxLDL) activates MAPK (ERK, JNK, p38) and nf-Kb pathways, which drive APC maturation and increase expression of MHC molecules and co-stimulatory receptors (CD80/CD86), enabling effective CD28-dependent T-cell activation (79),. Functionally, ERK supports antigen-processing capacity, whereas JNK and p38 modulate antigen-presentation programs; in chronic inflammatory settings, p38 can also favor regulatory outputs, including IL-10 production (80, 81). nf-Kb signaling sustains inflammatory cytokine release (e.g., TNF-α, IL-6), reinforcing APC activation and T-cell survival (82). Collectively, CD36-dependent APC activation shapes the cytokine milieu (including IL-12, IL-6, and TNF-α) to guide T-cell differentiation and coordinate adaptive immunity (82–84) (**Figure 3**).

### 5.2 T cell priming, polarization, and tolerance

CD36 shapes T-cell fate through both antigen-presenting cell (APC)–extrinsic effects (antigen uptake, processing, and costimulatory programming) and T-cell–intrinsic effects (lipid uptake and metabolic reprogramming). Consequently, its impact on T-cell priming and polarization is strongly context dependent.

In lipid-rich or pathogen-associated settings—for example, in the presence of oxLDL or microbial lipids—CD36 engagement on APCs often coincides with inflammatory cues (including Toll-like receptor signaling) that promote APC maturation and costimulation. Under these conditions, CD36-dependent antigen handling is frequently associated with enhanced effector polarization, particularly toward Th1 and Th17 programs (85).

By contrast, during efferocytosis under non-inflammatory or resolving conditions, CD36 preferentially supports a tolerogenic APC phenotype, favoring the induction of FoxP3^+^ regulatory T cells (Tregs) and the maintenance of peripheral tolerance (86). Mechanistically, CD36-mediated recognition of apoptotic cells activates anti-inflammatory pathways that increase TGF-β and IL-10 production (87). These cytokines suppress effector T-cell expansion and inflammatory cytokine output while promoting Treg differentiation and function (88, 89).

Beyond its effects mediated through APCs, CD36 also exerts T cell–intrinsic metabolic control. CD36 is expressed on multiple T-cell subsets, including CD8^+^ effector T cells and Tregs, particularly in lipid-rich microenvironments such as tumors and chronically inflamed tissues. As a high-affinity transporter for long-chain fatty acids and oxidized lipids, CD36 directly influences intracellular lipid availability, redox balance, and mitochondrial function (90). In CD8^+^ T cells, excessive CD36-mediated lipid uptake promotes lipid peroxidation and ferroptosis, resulting in impaired cytokine production and reduced cytotoxic capacity, where CD36 functions as an immunometabolic checkpoint that dampens antitumor immunity (85). In contrast, Tregs preferentially exploit CD36-dependent fatty-acid uptake and β-oxidation to sustain mitochondrial fitness, FoxP3 stability, and suppressive function (91).

Seemingly contradictory observations regarding CD36 function can therefore be reconciled by distinguishing where CD36 is expressed and which signals dominate the microenvironment. CD36 on APCs can facilitate effector T-cell priming when coupled to strong inflammatory and costimulatory signals, whereas CD36 on T cells directly modulates metabolic fitness and survival. In lipid-rich environments, CD36-driven lipid uptake may suppress CD8^+^ effector responses through oxidative stress and ferroptosis (92), while simultaneously reinforcing Treg-mediated immune regulation or tolerogenic antigen repertoires when antigen encounter occurs in the absence of robust danger signals (93).

### 5.3 Interaction with B cells and antibody responses

Although less extensively characterized, CD36 also plays an indirect role in shaping humoral immunity. By influencing antigen clearance and modulating cytokine production in APCs, CD36 can impact B cell activation and the dynamics of germinal centers, which are critical for the production of antibodies (94). There has been proposed cross-talk between CD36-mediated antigen uptake and Fc receptor (FcR) signaling, suggesting that CD36 may influence antibody class switching and potentially contribute to autoantibody generation (95). These interactions highlight the potential role of CD36 in autoimmunity as well as in protective antibody responses.

## 6 CD36 in inflammation: a double-edged sword

The dual role of CD36 in inflammation is dictated by its ability to engage various signaling pathways, yielding distinct outcomes based on the cellular context and the specific ligands involved. This section delineates how CD36’s signaling pathways diverge to promote both pro-inflammatory and anti-inflammatory responses, reinforcing the receptor’s versatile role in immune modulation.

### 6.1 Pro-inflammatory pathways triggered by CD36

**Figure 4:**
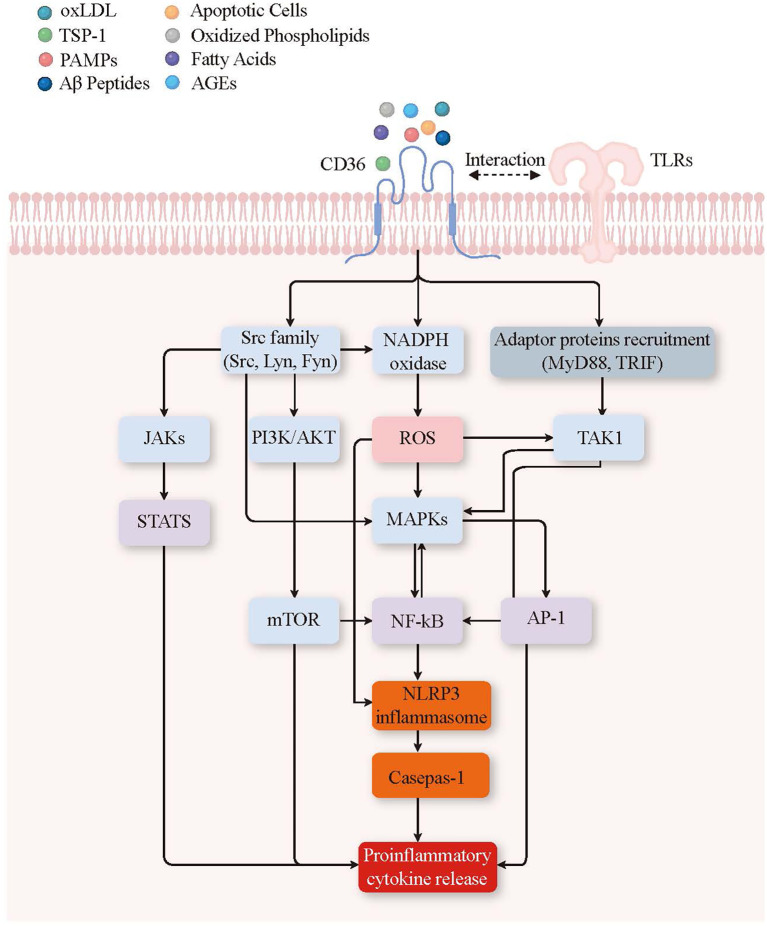
CD36 ligand and signal transduction pathways leading to proinflammatory cytokine release. Binding of various ligands (oxLDL, TSP-1, PAMPs, Aβ peptides, apoptotic cells, oxidized phospholipids, fatty acids, AGEs) to CD36 on the plasma membrane triggers the recruitment of TLRs, forming a complex that initiates several downstream signaling pathways. Key signaling components include kinases and signaling enzymes such as the Src family (Src, Lyn, Fyn), NADPH oxidase, JAKs, PI3K/AKT, TAK1, mTOR, and MAPKs (blue); second messengers and reactive molecules like ROS (pink); transcription and nuclear factors such as STATs, nf-Kb, and AP-1 (purple); inflammatory mediators including the NLRP3 inflammasome and caspase-1 (orange); and adaptor proteins such as MyD88 and TRIF (grey). These pathways converge to activate the NLRP3 inflammasome and caspase-1, leading to the release of pro-inflammatory cytokines, thereby promoting inflammation. Oxidized Low-Density Lipoprotein (oxLDL), Thrombospondin-1 (TSP-1), Pathogen-Associated Molecular Patterns (PAMPs), Amyloid-Beta Peptides (Aβ Peptides), Advanced Glycation End-products (AGEs), Non-receptor Tyrosine Kinases (Src family), including Proto-Oncogene Tyrosine-Protein Kinase Src (Src), Tyrosine-Protein Kinase Lyn (Lyn), Proto-Oncogene Tyrosine-Protein Kinase Fyn (Fyn), Janus Kinases (JAKs), Signal Transducers and Activators of Transcription (STATs), Phosphoinositide 3-Kinase/Protein Kinase B (PI3K/AKT), Reactive Oxygen Species (ROS), Nicotinamide Adenine Dinucleotide Phosphate Oxidase (NADPH oxidase), Mitogen-Activated Protein Kinases (MAPKs), Mechanistic Target of Rapamycin (mTOR), Nuclear Factor Kappa-Light-Chain-Enhancer of Activated B Cells (nf-Kb), Activator Protein 1 (AP-1), NOD-like Receptor Family Pyrin Domain Containing 3 Inflammasome (NLRP3 inflammasome), Caspase-1, Transforming Growth Factor Beta-Activated Kinase 1 (TAK1), Myeloid Differentiation Primary Response 88 (MyD88), and TIR-Domain-Containing Adapter-Inducing Interferon-β (TRIF).

Upon binding to ligands such as oxLDL, anionic phospholipids, and advanced glycation end products (AGEs), CD36 activates multiple signaling cascades. The proinflammatory signaling pathway triggered by CD36 and its ligand has been shown (**Figure 4**). Key among these is the MAPK pathway, involving ERK, JNK, and p38 MAPK, which facilitates the nuclear translocation of transcription factors such as nf-Kb and AP-1 (96). This translocation upregulates inflammatory genes like TNF-α, IL-6, and IL-1β (97), which are crucial in inflammatory disorders such as rheumatoid arthritis where CD36-mediated MAPK activation exacerbates tissue damage (98).

Further, CD36’s interaction with oxLDL and lipopolysaccharide (LPS) robustly stimulates the PI3K/AKT pathway, enhancing cellular responses that perpetuate inflammation, as observed in chronic conditions like COPD (99). Similarly, activation of the JAK/STAT pathway by CD36, especially in the context of systemic lupus erythematosus (SLE), exacerbates chronic inflammation through enhanced cytokine production (100). Additionally, the interaction of CD36 with TSP-1 initiates the recruitment of adaptor proteins like MyD88 and TRIF (101), leading to the activation of kinase TAK1 and NADPH oxidase, which results in the production of reactive oxygen species (ROS) (102, 103). NADPH oxidase generates ROS, which further activates MAPK pathways and nf-Kb, intensifying the inflammatory signaling cascades and contributing to neuroinflammatory processes in diseases like Alzheimer’s (104).

Moreover, CD36 activation can lead to the formation of the NLRP3 inflammasome, a multiprotein complex that plays a pivotal role in innate immunity (105). This inflammasome activates caspase-1, an enzyme critical for the maturation and release of pro-inflammatory cytokines such as IL-1β and IL-18 (106, 107). The activation of NLRP3 and caspase-1 contributes significantly to the inflammatory response, linking CD36 signaling to the exacerbation of inflammation in various chronic conditions such as type 2 diabetes (108).

### 6.2 Anti-inflammatory pathways triggered by CD36

**Figure 5:**
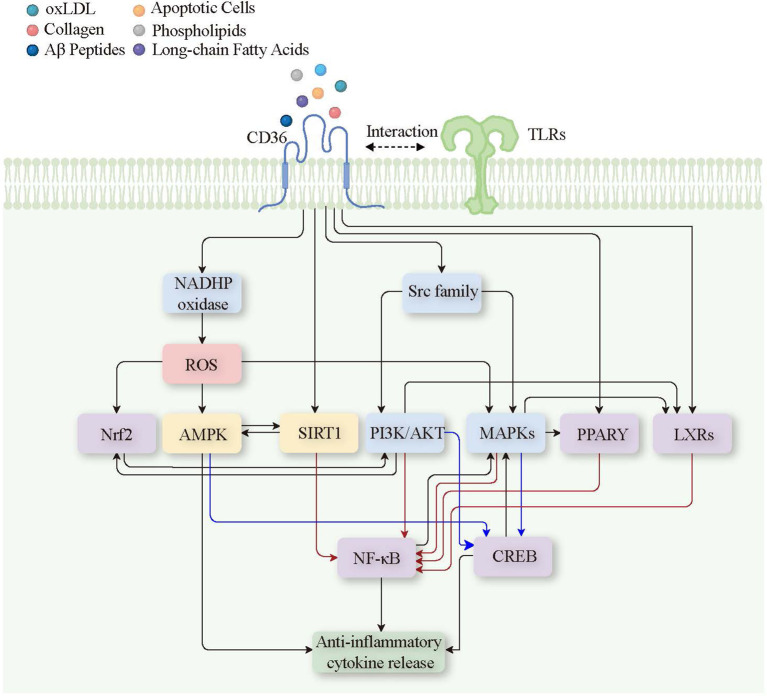
CD36 ligand and signal transduction pathways leading to anti-inflammatory cytokine release. Binding of various ligands (oxLDL, collagen, Aβ peptides, apoptotic cells, phospholipids, long-chain fatty acids) to CD36 on the plasma membrane triggers the recruitment of TLRs, forming a complex that initiates several downstream signaling pathways. Key signaling components include kinases and signaling enzymes such as NADPH oxidase, Src family, PI3K/AKT, and MAPKs; second messengers and reactive molecules like ROS; metabolic and stress response regulators such as AMPK and SIRT1; transcription factors and nuclear receptors including Nrf2, PPARγ, LXRs, nf-Kb, and CREB. These pathways converge to activate anti-inflammatory responses, leading to the release of anti-inflammatory cytokines, thereby promoting inflammation resolution. Red lines indicate the inhibition of nf-Kb, while blue lines indicate the activation of the CREB signaling pathway. Oxidized Low-Density Lipoprotein (oxLDL), Amyloid-Beta Peptides (Aβ Peptides), Toll-Like Receptors (TLRs), Nicotinamide Adenine Dinucleotide Phosphate Oxidase (NADPH oxidase), Reactive Oxygen Species (ROS), Nuclear Factor Erythroid 2–Related Factor 2 (Nrf2), AMP-Activated Protein Kinase (AMPK), Sirtuin 1 (SIRT1), Phosphoinositide 3-Kinase/Protein Kinase B (PI3K/AKT), Mitogen-Activated Protein Kinases (MAPKs), Peroxisome Proliferator-Activated Receptor Gamma (PPARγ), Liver X Receptors (LXRs), Nuclear Factor Kappa-Light-Chain-Enhancer of Activated B Cells (nf-Kb), and cAMP Response Element-Binding Protein (CREB).

Conversely, CD36’s engagement with anti-inflammatory ligands such as omega-3 fatty acids activates nuclear receptors like Liver X Receptors (LXRs) (109) and Peroxisome Proliferator-Activated Receptor gamma (PPARγ) (110). These receptors facilitate the transcription of genes that not only enhance cholesterol efflux but also diminish the expression of pro-inflammatory genes, supporting the resolution of inflammation and aiding tissue recovery (111). The anti-inflammatory signaling pathway triggered by CD36 and its ligand has been shown (**Figure 5**).

Activation of AMP-activated protein kinase (AMPK) by CD36, in response to its interaction with ligands, downregulates nf-Kb signaling, thereby reducing the production of pro-inflammatory cytokines (112, 113). This modulation is particularly advantageous in inflammatory bowel disease (IBD), where it helps mitigate intestinal inflammation and promotes mucosal healing (114). Additionally, CD36-mediated activation of the cAMP Response Element-Binding Protein (CREB) pathway increases the production of anti-inflammatory cytokines such as IL-10, crucial for suppressing inflammation in conditions like psoriasis (115).

Furthermore, engagement of CD36 with its ligands also triggers the Nrf2 pathway, a critical regulator of cellular defense mechanisms against oxidative stress (116), which is particularly significant in conditions where inflammation results in tissue damage, such as multiple sclerosis (MS) (117). Enhancing this pathway through CD36 activation can substantially mitigate disease progression and severity by boosting cellular resilience against oxidative stress (118).

Additionally, CD36 activation leads to the stimulation of SIRT1 (Sirtuin 1), a NAD+-dependent deacetylase that plays a key role in cellular stress responses (119, 120). SIRT1 activation results in the deacetylation and inhibition of nf-Kb, further contributing to the reduction of pro-inflammatory cytokine production (121). This pathway also enhances mitochondrial function and promotes autophagy, aiding in the resolution of inflammation and the maintenance of cellular homeostasis (122, 123).

### 6.3 Underlying mechanisms of CD36 driving both pro-inflammatory and anti-inflammatory pathways

CD36 orchestrates diverse immune responses by activating both pro-inflammatory and anti-inflammatory pathways, reflecting its complex regulatory role. This section explores the mechanisms enabling these dual actions, focusing on cellular context, ligand specificity, signaling overlap, transcriptional regulation, and feedback loops.

#### 6.3.1 Cellular context

The impact of CD36 signaling is highly dependent on the cellular environment, which varies based on cell type and activation state. This variability affects how ligands interact with CD36, influencing downstream signaling pathways. For instance, in macrophages, CD36 engagement with oxLDL typically promotes a pro-inflammatory response, facilitating the formation of foam cells, a key process in atherosclerosis development (124). In contrast, when endothelial cells engage CD36 with the same oxLDL under stress-free conditions, it triggers protective, anti-inflammatory pathways that help maintain vascular homeostasis (125). This difference can be attributed to the distinct sets of downstream signaling molecules and receptors expressed in macrophages versus endothelial cells, illustrating how the cellular context dictates the outcome of CD36-ligand interactions (126).

#### 6.3.2 Ligand specificity and receptor crosstalk

Ligands may not be exclusively pro-inflammatory or anti-inflammatory. For example, phospholipids, commonly recognized for their role in membrane structure and pro-inflammatory signaling (127), can potentially activate anti-inflammatory pathways under certain conditions, such as low concentration exposure or in the presence of other modulating signals (128). This dual potential is often due to receptor crosstalk. Receptor crosstalk occurs when the binding of a ligand to CD36 influences the activity of other receptors or signaling pathways (90). For instance, binding of phospholipids to CD36 may modulate the function of TLRs or other scavenger receptors, leading to a combination of signaling responses (129). This interaction can result in the activation of anti-inflammatory pathways through receptors like LXRs or PPARγ (130), while simultaneously influencing pro-inflammatory pathways through TLRs or nf-Kb (131). The complexity of these interactions results in a mixed response, where the final outcome depends on the balance and context of these signaling events.

#### 6.3.3 Signaling pathway overlap

The pathways leading to inflammatory responses often share common molecular components with those leading to anti-inflammatory outcomes. For instance, both pro-inflammatory and anti-inflammatory pathways might involve MAPKs and PI3K/AKT (132) but diverge at a certain point where specific adapters or transcription factors such as nf-Kb (pro-inflammatory (133)) versus PPARγ and LXRs (anti-inflammatory (134)) are activated. The initial segments of these pathways may be similar, which allows a ligand the potential to influence both pathways depending on the dynamics within the cell at the time of activation.

#### 6.3.4 Differential regulation of transcription factors

Some transcription factors can have dual roles depending on the context, such as nf-Kb, which is typically associated with inflammation but can also participate in the resolution phase under certain conditions (135). For instance, during the later stages of inflammation, nf-Kb can induce the expression of anti-inflammatory genes like IL-10 and TGF-β in the presence of specific cofactors or signaling molecules (136). Additionally, in the context of chronic inflammation, such as in atherosclerosis, nf-Kb activation in regulatory T cells (Tregs) can promote the production of anti-inflammatory cytokines, aiding in the resolution of inflammation (137). The balance between different forms of the same transcription factor or its interaction with other cofactors can shift the response from pro-inflammatory to anti-inflammatory, illustrating the complexity and adaptability of immune responses.

#### 6.3.5 Feedback mechanisms

Cells often have intrinsic feedback mechanisms that can alter the response to a stimulus. For example, prolonged exposure to an inflammatory stimulus might activate regulatory pathways aimed at suppressing overactive inflammatory responses to prevent tissue damage, thus engaging anti-inflammatory mechanisms even in the presence of a primarily pro-inflammatory ligand (138).

## 7 Therapeutic targeting of CD36

CD36 is a multifunctional scavenger receptor that integrates lipid uptake with inflammatory and immune signaling. Because it participates in diverse processes (e.g., fatty acid transport, oxLDL recognition, macrophage activation, and immune-cell metabolic programming), CD36-targeted interventions must be context-specific (cell type, ligand milieu, and disease stage). Therapeutic development is therefore evolving beyond broad CD36 inhibition toward modality- and tissue-tailored strategies that (i) reduce CD36 expression or activity in pathogenic compartments, (ii) block defined CD36-ligand axes driving disease, (iii) rewire CD36 trafficking/signaling to avoid systemic metabolic liabilities, and (iv) use targeted delivery or rational combinations to improve efficacy and safety.

### 7.1 Modulating CD36 expression and activity

To modulate CD36 pathways, researchers increasingly apply nucleic-acid based approaches that enable cell- or tissue-selective control of CD36 function. These include RNA interference (RNAi) and antisense strategies to reduce CD36 expression, as well as CRISPR/Cas9-based editing to generate durable loss-of-function in experimental models (139). Importantly, delivery technologies (e.g., lipid nanoparticles and ligand-directed carriers) are enabling *in vivo* editing or transcriptional silencing in disease-relevant tissues, which may mitigate systemic effects. Recent work also highlights that targeting upstream regulators (e.g., transcriptional programs that govern FA uptake and scavenger receptor expression) can indirectly tune CD36 activity while preserving basal homeostasis (13). Beyond direct receptor knockdown, emerging strategies include modulating downstream metabolic nodes (e.g., AMPK activation) or inflammatory programs to reduce CD36-driven lipid overload and sterile inflammation (140).

### 7.2 Inhibiting CD36-ligand interactions

Directly blocking pathogenic CD36–ligand interactions is a key therapeutic strategy. While small-molecule inhibitors such as sulfosuccinimidyl oleate (SSO) suppress CD36-mediated fatty acid uptake and signaling, their irreversible action and limited selectivity hinder translation (141), motivating efforts to develop reversible, more selective antagonists and define ligand-specific binding determinants. In parallel, biologics (peptides/antibodies) that target defined CD36 epitopes have advanced; the peptide EP80317 reduced atherosclerosis in ApoE-deficient mice (142). In oncology, CD36 is emerging as an immunometabolic checkpoint: the humanized IgG4 antibody PLT012 enhanced antitumor immunity in lipid-rich tumors (143).

### 7.3 Targeting CD36 trafficking and signaling bias

An emerging concept is that CD36 pathogenicity can be reduced without complete receptor ablation by controlling receptor localization, turnover, and signaling output. CD36 trafficking is dynamically regulated by post-translational modifications, including palmitoylation, which influences membrane residence and endocytosis (144). Modulating these processes can, in principle, reduce deleterious lipid uptake or inflammatory signaling in specific compartments while maintaining essential basal functions. For example, inhibition of CD36 palmitoylation was reported to promote mitochondrial localization, enhance fatty acid beta-oxidation, and alleviate lipid accumulation and inflammation in a NASH model (145). Such strategies point to a next generation of CD36-directed therapies that target receptor behavior (trafficking/signaling bias) rather than simply receptor abundance.

### 7.4 Precision delivery and rational combination strategies

Given CD36’s broad expression, precision delivery has become central to improving therapeutic index. Nanocarrier-based systems and targeted nanoparticles can concentrate CD36 inhibitors within disease-relevant tissues such as atherosclerotic plaques, inflamed adipose depots, fibrotic liver, or the tumor microenvironment (146). Targeting ligands (antibodies, peptides, aptamers) and stimuli-responsive formulations (pH/enzymatic triggers) offer additional specificity. Combination regimens are also gaining traction: CD36 blockade can be paired with lipid-lowering agents or anti-inflammatory drugs in metabolic disease, and with immune checkpoint inhibitors or anti-angiogenic therapy in oncology, where preclinical data suggest CD36 targeting may restore responsiveness to PD-1/PD-L1 blockade in lipid-rich, immune-excluded settings (147).

### 7.5 Challenges and potential side effects of targeting CD36

#### 7.5.1 Complex role in physiology and immunity

Targeting CD36 poses significant challenges due to its involvement in crucial physiological processes such as lipid metabolism, clearance of apoptotic cells, and immune response regulation. Because CD36 facilitates these essential functions, inhibiting it could disrupt normal cellular operations, potentially leading to unintended consequences (148). For instance, impaired clearance of apoptotic cells due to CD36 inhibition might result in the accumulation of cellular debris, provoking chronic inflammation (149).

#### 7.5.2 Risk of exacerbating inflammatory conditions

Another major concern with targeting CD36 is the risk of exacerbating chronic inflammatory conditions. CD36 plays a critical role in balancing pro-inflammatory and anti-inflammatory signaling pathways. In conditions like atherosclerosis or metabolic syndrome, where inflammation is a central feature, indiscriminate inhibition of CD36 could shift the balance towards increased inflammation, potentially worsening the clinical outcomes (29).

#### 7.5.3 Selective inhibition challenges

The broad expression of CD36 across various tissues and its interaction with multiple ligands complicates the development of targeted therapies. Selective inhibition of CD36 is essential to avoid interfering with its beneficial effects, such as its roles in clearing oxidized lipoproteins and apoptotic cells (150). Developing receptor-specific modulators that can selectively block pathological interactions without impeding the receptor’s normal functions remains a crucial area of research.

#### 7.5.4 Targeted delivery systems

To mitigate the potential side effects of systemic CD36 inhibition, research is focusing on targeted delivery systems. These systems aim to concentrate therapeutic agents at specific disease sites, such as atherosclerotic plaques or tumor tissues, minimizing systemic exposure and reducing adverse effects on healthy tissues. Advanced drug delivery technologies, including nanoparticle-based carriers and localized drug depots, offer promising methods to achieve this targeted approach (146).

## 8 Conclusion and perspectives

Throughout this review, we have detailed the multifunctional role of CD36, emphasizing its critical influence in both promoting and regulating immune responses. CD36’s involvement in key processes such as phagocytosis, inflammation, and the integration of innate and adaptive immune systems underscores its potential as a valuable therapeutic target across a spectrum of diseases. By facilitating essential interactions within the immune system, CD36 plays a pivotal role in the progression of metabolic disorders and cardiovascular diseases.

The intricate role of CD36 in immune modulation is vital for developing innovative therapeutic strategies. Its impact on the functionality of APCs and T-cell responses presents strategic opportunities for enhancing immune effectiveness, which is particularly valuable in improving vaccine efficacy. Future research focusing on CD36 pathways holds promise for not only mitigating chronic inflammation but also for preventing the severe complications associated with inflammatory conditions. By expanding our understanding and targeting of CD36, we can potentially revolutionize treatment paradigms, improving outcomes across a broad range of immune-related disorders.

## Data Availability

The original contributions presented in the study are included in the article/supplementary material. Further inquiries can be directed to the corresponding authors.
